# Enhanced anti-inflammatory and antibacterial efficacy of a novel gum ghatti-infused polymeric film for ocular drug delivery

**DOI:** 10.5599/admet.3200

**Published:** 2026-05-28

**Authors:** Swagatika Das, Sk Habibullah, Yashwant Giri, Amulyaratna Behera, Gurudutta Pattnaik, Biswaranjan Mohanty

**Affiliations:** 1 Department of Pharmaceutics, School of Pharmacy and Life Sciences, Centurion University of Technology and Management, Odisha 752050, India; 2 Department of Pharmaceutics, University Department of Pharmaceutical Sciences, Utkal University, Vani Vihar, Bhubaneswar, Odisha 751004, India; 3 Department of Pharmaceutics, School of Pharmacy, DRIEMS University, Tangi, Cuttack, Odisha 754022, India

**Keywords:** Corneal integrity, inflammation, irritation, transparency

## Abstract

**Background and purpose:**

Ocular inflammation is a key challenge after ocular surgery, and resistance has developed in the bacterial strain. Primarily, the short residence time of the ocular formulation makes it more effective in treating and alleviating inflammation.

**Experimental approach:**

In this study, a moxifloxacin-incorporated gum ghatti-infused poly(vinyl alcohol-chitosan) polymeric film was developed by solvent casting. Various properties of the ocular composite film, including mucoadhesiveness, strength profile, and crystal size, were evaluated.

**Key results:**

The prepared composite film exhibited a red-yellow hue. XRD and DSC analyses revealed that the drug in the formulation is amorphous. The release profile reduces the release rate and follows both Fickian- and non-Fickian-mediated pathways. A clear zone of inhibition was observed against both Gram-negative (*Pseudomonas aeruginosa*) and Gram-positive (*Staphylococcus aureus*) bacteria. The in vivo anti-inflammatory potential of carrageenan was observed against the prepared formulations, and within 1.5 hours, redness and inflammation diminished. Corneal integrity was maintained, with no dark spots observed in the corneal region, confirming that no coagulant or thrombosis of blood occurred in the eye.

**Conclusion:**

Based on the foregoing discussion, the formulation shows potential for treating ocular conjunctivitis and can be safely used for pink eye, bacterial conjunctivitis, or allergic conjunctivitis.

## Introduction

Ocular inflammation poses a significant challenge in ophthalmology, encompassing a wide range of inflammatory diseases [1,2]. These symptoms may affect various ocular structures, including the conjunctiva, cornea, sclera, orbit, adnexa, and ocular surface [3]. The occurrence of such symptoms can lead to conjunctivitis, uveitis, and keratitis [4], which may cause discomfort, vision impairment, and even permanent damage if left untreated [5]. Such diseases can be treated with traditional therapies, including topical corticosteroids and nonsteroidal anti-inflammatory drugs (NSAIDs). However, these medications frequently cause adverse effects, including ocular surface toxicity and elevated intraocular pressure [6,7]. Consequently, there is a need to develop new treatment methods that improve drug delivery to the eye while reducing side effects. In this context, polymeric films have emerged as a promising option due to their ability to minimize systemic absorption, increase corneal contact time, and enable continuous medication delivery [8,9].

In recent years, poly(vinyl alcohol) (PVA) and chitosan (CS) have emerged as biocompatible polymers with significant potential for various biomedical applications [10]. In combination, they create a composite material that is both diverse and promising, with distinct features that make it a desirable option for developing eye therapy modalities. PVA is a synthetic, water-soluble, non-toxic polymer with properties that make it an excellent choice for emulsifying and film-forming applications. Additionally, PVA's high elastic strength, inertness, and flexibility have enabled its application in food packaging and medical delivery devices. PVA can enhance the compatibility of biopolymers within the chitosan-starch film structure, thereby improving mechanical properties [11]. In a related work, Abraham et al. (2016) showed that combining PVA and chitosan with a cross-linking agent can enhance the films' tensile and thermal characteristics [12]. CS is an ideal biopolymer for film fabrication for biomedical applications owing to its biodegradability, biocompatibility, and non-toxicity. However, CS is a rigid-chain polymer, yielding stiff films that readily rupture or tear in real-world conditions [12,13]. The most effective method for enhancing the performance of CS films under these conditions is to combine them with a polar biodegradable polymer, such as PVA. Furthermore, CS exhibits notable antibacterial activity, making it a viable material for a range of pharmaceutical, food, and medical applications. Chitosan's distinct chemical structure and interactions with microbial cells are responsible for its antibacterial effect [14].

This work in the emerging field focuses on developing and testing a new ocular drug delivery system using PVA and CS films infused with gum ghatti (GG). GG, a natural biopolymer and Indian gum, is widely used in food, pharmaceutical, and other industries for its excellent thickening and emulsifying properties [15]. It is a complex non-starch polysaccharide composed of D-mannose, D-galactose, L-arabinose, and D-glucuronic acid [16]. GG was incorporated into the PVA-CS matrix to enhance mucoadhesion, ocular retention, and the duration of drug release. Its biocompatibility and biodegradability make it suitable for ocular delivery [17,18]. Subsequently, we described the film formulation process and the characterization methods used to evaluate its properties. The drug-release study used moxifloxacin as a model drug, selected for its proven effectiveness against bacterial eye infections such as keratitis and conjunctivitis and its potential anti-inflammatory effects [19]. This comprehensive analysis will elucidate the therapeutic potential of our innovative ocular medication-delivery technology to reduce inflammation and promote ocular health.

## Experimental

### Materials and methods

Poly(vinyl alcohol) and low-molecular-weight chitosan (<100 kDa) were procured from Central Drug House (P) Ltd., Mumbai, India, while glutaraldehyde was purchased from NICE Chemicals (P) Ltd., Kochi, India. Nutrient agar and gum ghatti were purchased from Himedia (P) Ltd., Mumbai, India. Moxifloxacin was provided as a gift by Alkem Laboratories, and double-distilled water was used throughout the experiment.

### Film-forming solutions and their preparation

10 % poly(vinyl alcohol) (10 g PVA in 90 g water) was prepared using an overhead stirrer (Q-19 A, Remi Elektrotechnik Limited, Kolkata, India). A CS solution (5 wt.%) was prepared by blending acetic acid and water (1:1) and stirring at room temperature for 8 hours. Different concentrations of GG solution (1, 2.5, 5 and 10 wt.%) were prepared by dissolving GG in distilled water and stirring until a clear solution formed. All solutions were then filtered through nylon cloth. All polymeric solutions were mixed as specified in Table 1. PVA/CS/GG solutions were prepared using an overhead mechanical stirrer (400 rpm, 20 min). Meanwhile, drug-containing films were made by mixing moxifloxacin into the film-forming solution until complete dissolution. In the final step, the crosslinker (glutaraldehyde solution: distilled water, 0.5:9.5) was added and the mixture was homogenized for 2 minutes. Then, the solution (20 mL) was poured into a 90-mm Petri dish and placed in a hot-air oven at 40 °C. After drying, the composite was packed in a sealed zippered container for subsequent use.

**Table 1.** Composition of the PVA/CS/GG ocular films (volume of cross-linking solution 1 mL)

Formulation code	Content, g			Amount of moxifloxacin HCl, mg cm^-2^
	PVA solution (10 wt.%)	CS solution (5 wt.%)	GG solution	
PCG0	14	6	0	-
PCG1	12	6	2^a^	-
PCG2	12	6	2^b^	-
PCG3	12	6	2^c^	-
PCG4	12	6	2^d^	-
PCG0D	14	6	0	5
PCG1D	12	6	2^a^	5
PCG2D	12	6	2^b^	5
PCG3D	12	6	2^c^	5
PCG4D	12	6	2^d^	5

Concentrations of GG solution: ^a^1, ^b^2.5, ^c^5 and ^d^10 wt.%

### Physicochemical properties of the fabricated composites

Various physicochemical properties, including weight uniformity, ocular composite thickness, surface pH, opacity (Equation 1), folding endurance, and blood biocompatibility (Equation 2), were measured.



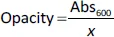

(1)


where *x* / nm represents the thickness of the films in mm and Abs_600_ represents the absorbance at 600 nm.



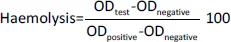

(2)


where OD_test_ is the absorbance of the tested film sample at 545 nm, OD_negative_ refers to the absorbance of the negative control at 545 nm and OD_positive_ is the absorbance of the positive control at 545 nm.

### Transparency

Transparency of the ocular composites was measured both using a ruler and spectrophotometrically. Gently, the ruler was placed under the fabricated films, and photographs were taken. Visually, the number on the ruler was visible through the fabricated films. To assess the radiation effect and UV rays transmittance, the films were cut into 40×50 mm pieces, and transmittance was recorded from 200 to 800 nm.

### Microscopy

Optical images were captured using a compound microscope equipped with a CCD camera (Magnus MX21i LED, Japan). A small piece of film was cut and placed on the slide, and microarchitecture was observed. The surface morphology of the prepared composite was examined using field-emission scanning electron microscopy (JEOL JSM-6510, Japan) at higher magnification [20].

### Colour analysis

The effects of CS and GG on the colour coordinates were observed visually. To analyse the hue system in the prepared ocular film, the colour variation of the fabricated films was measured using a colourimeter (Premier Colorscan, SS 5100H, Mumbai, India). The measurement was performed in direct transmittance mode using the CIE 1976 L* (lightness), a* (+a redness or -a greenness) and b* (+b yellowness or -b blueness) colour space. Using the L*, a* and b* coordinates, colour difference (Δ*E*), whiteness (WI) and yellowness index (YI) were calculated using Equations (3), (4) and (5), respectively [21].



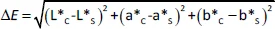

(3)




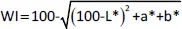

(4)




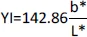

(5)


L*_C_, a*_C_, b*_C_ are the values of the control film parameters (ocular film without gum ghatti), whereas the numerical values obtained for different GG-films were substituted with symbols having an ‘s’ tag.

Swelling profile

The swelling profile of the PVA/CS/GG was evaluated by cutting it into 20×20 mm squares and weighing the squares. Pre-weighted films were immersed in artificial tear fluid (ATF; pH 7.4) at room temperature. Briefly, the pre-weighted ocular film was submerged in a petri dish containing 2 mL of ATF. At predetermined time intervals, the film was carefully removed, and excess ATF was wiped off with tissue paper. The film weight was recorded, and the process was repeated until three consecutive measurements were identical. The swelling ratio was calculated using Equation (6).



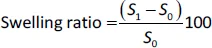

(6)


Where *S*_0_ and *S*_1_ represent the sample weight before and after absorption of ATF, respectively.

### Molecular and mechanical evaluation

FTIR, DSC and XRD analyses of the prepared composite film should be performed using the methods described by the respective authors [22].

Mucoadhesion of the PVA/CS/GG ocular film was tested with a CTX texture analyser (Brookfield, Ametek, United States). A 35 mm cylindrical probe equipped with a 5 kg load cell was used to assess adhesive properties and was applied to the gelatine substrate (6.67 wt.%). 1 mL of ATF was evenly spread over the gelatine surface to mimic the ocular mucosa. The film was cut into a circular disc and attached to the probe tip. The probe was pressed onto the gelatine gel in a glass beaker with a force of 0.5 N for 120 seconds. Various mucoadhesion parameters, such as peak adhesion force (PAF), total work of adhesion (TWA), and cohesiveness, were measured and recorded using the built-in Texture Pro software.

To evaluate the breaking force of the ocular composite, prepared films were cut into 20×20 mm pieces, placed between two rings, and secured. A 4 mm-diameter cylindrical sensor (TA44) was used in the experiment. Throughout the experiment, the probe velocity was fixed at 2.00 mm s^-1^.

### In vitro drug diffusion study

Drug diffusion analysis was performed using the method described by the various authors [23]. A modified Franz diffusion cell was used for the study. A film was cut into 10×10 mm and placed on the pre-hydrated dialysis membrane (soaked overnight in artificial tear fluid, ATF), which served as a semipermeable barrier separating the donor and receptor compartments. The temperature of the receptor compartment was maintained at 37±1 °C with continuous stirring at 100 rpm throughout the experiment. At a predetermined time interval, 1 mL of the sample was withdrawn and replaced with fresh ATF solution. The concentration of moxifloxacin in the withdrawn solution was measured using a UV-vis spectrophotometer (UV-1900i, Shimadzu Corporation, Japan) at 289 nm.

### MTT-assay of the fabricated formulations

The MTT Assay was performed on a 96-well plate. Human corneal epithelial cells (HCECs) were used to evaluate the toxicity of the formulations. The test was conducted by the method described elsewhere [24]. In brief, HECE was incubated in a healthy environment (37 °C) for 24 hours. Inoculated HECE having 3000 to 4000 cells *per* well in 100 μL is taken as standard cell growth, and test samples were treated in 96-well plates after incubation. Test and reference samples were measured for absorbance at 570 nm in DMSO. Cell viability was determined as the percentage relative to the control value (set to 100%).

### Antimicrobial activities

The antimicrobial activity of the composite film was evaluated against both Gram-positive (*S. aureus*; ATCC 29213) and Gram-negative (*P. aeruginosa*; ATCC 27853) bacteria. Prepared films were placed on a freshly prepared agar well plate using the disc diffusion method. The plates were incubated for 24 h at 37 ± 1 °C. The zone of inhibition (ZOI) was measured with a ruler.

### Ocular irritation study

An ocular irritation test was conducted after obtaining approval from the Institutional Animal Ethics Committee, with protocol clearance number (CPCSEA-IAEC-IPT/04/23), dated 03 October 2023. During the experiment, ocular abnormality-free rabbits were used, and the study was conducted strictly in accordance with the ARRIVE guidelines and the Draize test protocol. A small piece of film was placed in the conjunctival sac of the left eye and considered the test group. The right eye was injected with 0.1 mL of normal saline and referred to as the control. After inserting the film at different time intervals, both eyes were examined for ocular abnormalities (redness, swelling, injury, reaction, or inflammation).

### In vivo ocular inflammation potential of the composite

Healthy rabbits were used to assess the in vivo anti-inflammatory activity of the prepared film. For this, ocular abnormalities and disease-free rabbits were selected and divided into three groups of three rabbits each. Group I served as the negative control and was treated with normal saline. Group II was treated with carrageenan (200 μL; 2 % w/v) and designated as the test group, and Group III was used as the standard group (treated with the fabricated film formulation after injecting carrageenan). Inflammation was induced by injecting carrageenan, and a sterilized composite film (UV-irradiated at 25 cm for 10 min) was placed in the cul-de-sac region of the eyes 1 hour after inflammation. After the conclusion of the experiment, fluorescein staining of the cornea was done to evaluate the damage to the epithelium. Moxifloxacin eye drops (MOXI TOR; 0.5%) were used to prevent infection and facilitate recovery.

### Statistical analysis

All experiments were conducted in triplicate, and results are presented as mean ± standard deviation (SD).

## Results and discussion

### Fabrication of the PVA/CS/GG composite film

**Figure 1.**
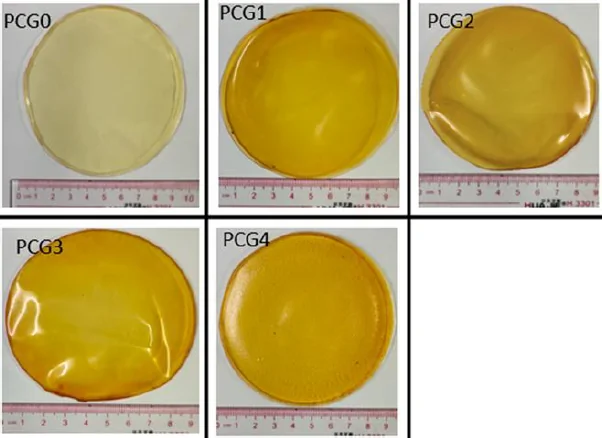
Fabricated PVA/CS/GG composite film formulations

PVA/CS/GG composite films loaded with moxifloxacin hydrochloride were prepared using the solvent evaporation method, as shown in Figure 1. This method is conventional and widely applicable for film preparation. Glutaraldehyde (GTA) was used as a crosslinker at a very low concentration, as documented earlier in the literature, for drug delivery [25]. Prepared films undergo a colour change from white to brownish-red as the chitosan concentration increases and chitosan interacts with gum ghatti. Prepared films were flexible in nature. Additionally, free GTA was neutralized by immersing it in a 0.1 M glycine solution for four hr. to prevent ocular chemosis or irritation [26].

### Physicochemical properties of the fabricated films

Various physicochemical parameters, including thickness, weight uniformity, pH, folding endurance, opacity, and blood compatibility, were assessed and are tabulated in Table 2. The film thickness was lowest at 0.16 mm (PCG0) and highest at 0.26 mm (PCG4). A low standard deviation in thickness suggested uniform dispersion of the drug and polymeric particle throughout the film. pH of the film plays a vital role in ocular drug delivery. High or low pH may cause chemosis and irritation of ocular epithelial tissue, resulting in swelling and inflammation. The reported data indicated that the pH range of 5.5 to 7.5 was safe for ocular delivery. Our experimental results showed that the pH was within the safe range, indicating that the fabricated film formulation is suitable for ocular drug delivery. The prepared films exhibit a uniform weight distribution in increasing order, attributed to the increase in polymer concentration. Folding endurance was >200, indicating excellent flexibility. The film's opacity increased in an orderly manner, indicating that transparency decreased. Increasing the opacity may result in a hydrophilic interaction between gum ghatti and chitosan, leading to a yellowish-red colour. All fabricated formulations exhibited haemolysis of less than 5 %, indicating safety for corneal delivery.

**Table 2.** Physicochemical properties of the fabricated formulations

Formulation code	Thickness, mm	Specific weight, mg cm^-2^	Surface pH	Folding endurance	Hemolysis, %	Opacity
PCG0	0.16 ± 0.02	31.45 ± 0.45	6.45±0.27	>200	0.58 ± 0.11	0.62±0.05
PCG1	0.18 ± 0.02	33.03 ± 0.47	6.70±0.24	>200	0.30 ± 0.06	0.68±0.04
PCG2	0.21 ± 0.03	34.57 ± 0.50	6.76±0.17	>200	0.21 ± 0.07	0.68±0.05
PCG3	0.23 ± 0.07	35.23 ± 0.35	6.73±0.01	>200	0.38 ± 0.10	0.72±0.05
PCG4	0.26 ± 0.02	37.07 ± 0.25	6.73±0.20	>200	0.22 ± 0.05	0.84±0.03

### Transparency of the fabricated films

**Figure 2.**
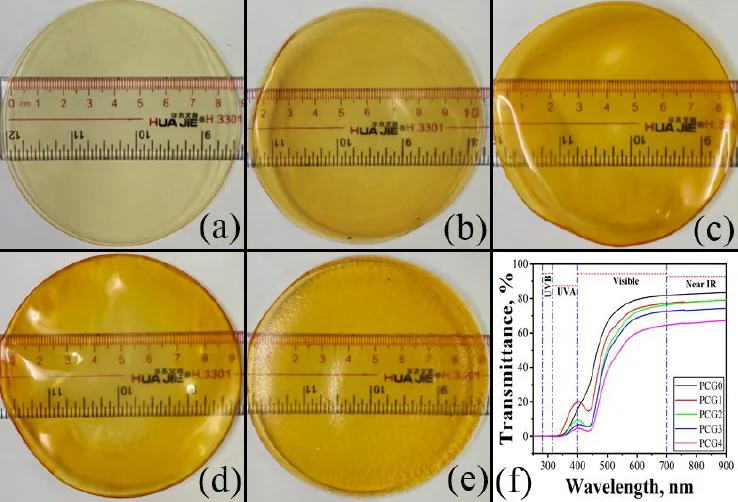
Transparency of the fabricated films. (a) PCG0, (b) PCG1, (c) PCG2, (d) PCG3, (e) PCG4 and (f) Transparency of the film through UV-Visible spectrophotometer

Transparency of the films was assessed using a ruler (Figures 2a-e) and a UV-Visible spectrophotometer (Figure 2g). The colour of the film changed from light white to yellowish-red, indicating an interaction between chitosan and gum ghatti. The addition of polymer changes the transparency of the fabricated film formulations. Transparency was recorded over the range of 280 to 900 nm using a UV-Visible spectrophoto-meter. The spectrum was divided into regions: 280 to 320 nm (UVB), 320 to 400 nm (UVA), 400 to 700 nm (visible), and 700 to 900 nm (near-IR). The average light transmission, % in the UV-visible region was found in the order of PCG0 (58.75) > PCG1 (54.31) > PCG2 (50.96) > PCG3 (48.15) > PCG4 (41.94). Various authors reported that the presence of chitosan reduced the transparency of the composite film [27,28]. In the UVB region, the highest light transmittance was found in PCG1, whereas the lowest was in PCG0. Rays in the UVA region (290 to 350 nm) are hazardous to human skin. It causes high tissue damage and chemosis. The average transparency in the UVA region was PCG0 (8.96), PCG1 (4.77), PCG2 (3.51), PCG3 (2.26), and PCG4 (1.52). PCG0 showed greater transparency in the visible region than the others. In the near IR regions, average transmittance was found to be 82.86, 78.25, 77.95, 73.52 and 65.97 % in PCG0 to PCG4, respectively. All the fabricated films exhibit good transparency and enhanced UV-shielding effect. Hence, the prepared film can be used for safe corneal delivery. The lower transmittance observed indicated the presence of gum ghatti interference with chitosan, decreasing its transparency. From this result, it was found that the presence of chitosan and gum ghatti reduces harmful UV radiation and enhances the UV-shielding effect, with an increase in gum ghatti concentration.

### Microscopic analysis

**Figure 3.**
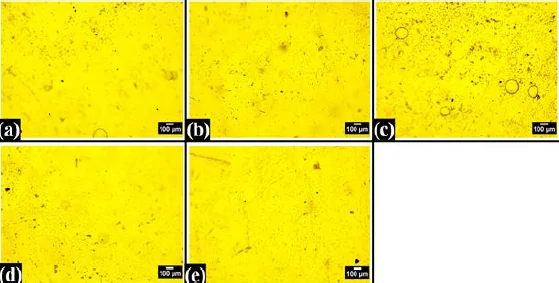
Brightfield micrographs of the fabricated formulations (a) PCG0, (b) PCG1, (c) PCG2, (d) PCG3 and (e) PCG4.

To investigate microarchitectural changes after the incorporation of gum ghatti, bright-field microscopy images were acquired and are shown in Figure 3. Bright-field micrographs revealed a smooth surface, except for PCG2. These micrographs show the scattering of small, dark particles across the surface. Some globular structures are also present in the micrographs, indicating interaction between the hydrophobic domains of PVA and chitosan and the hydrophilic domain of gum ghatti.

**Figure 4.**
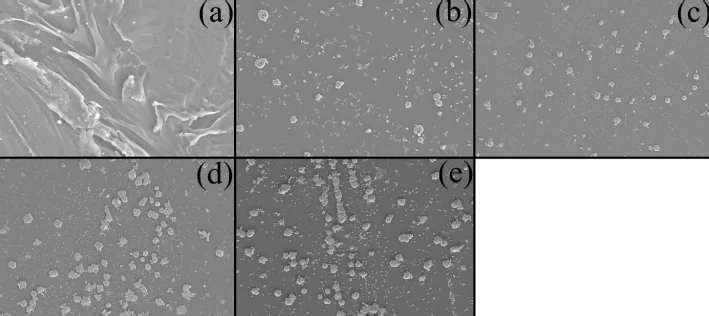
Fe-Sem images of the fabricated formulations (a) PCG0, (b) PCG1, (c) PCG2, (d) PCG3 and (e) PCG4

To improve understanding of the brightfield micrographs, images were acquired using a high-magnification field-emission scanning electron microscope. Figure 4 indicates the Field Emission Scanning Electron Microscopy (FE-SEM) images. Figure 2a represents the images of PCG0. The macromolecular structure of chitosan was present on the film surface, with some white dots. The white dots may represent the agglomeration of CS particles with PVA molecules. Upon incorporation of gum ghatti, the molecular flexibility of chitosan could be lost or hindered within GG. This may be due to the presence of the crosslinker and to larger interchain distances within the polymer network, which aggregate the bipolymeric structure and enhance adhesion between the two phases.

Figure 4 shows concentration-dependent scattering of semicrystalline structure across the surface. A smooth surface indicates that crosslinkers (GTA) reduced interfacial tension between hydrophobic and hydrophilic regions within the polymer matrix. The small black spot or particle may be due to chitosan. A similar observation was reported by Qureshi *et al.* [29]. Verma *et al.* [30] prepared pectin and gum ghatti hydrogel. Their results showed that a sharp, semicrystalline, and porous morphological structure provides a large surface area for adsorption [30]. A similar observation was reported by Narasagoudr *et al.* [31]. The researcher prepared a composite film with guar gum/chitosan and a gum ghatti polymeric film and observed similar aggregation. Aggregation of the polymeric gum and PVA may improve mechanical properties.

### Colour analysis

**Figure 5.**
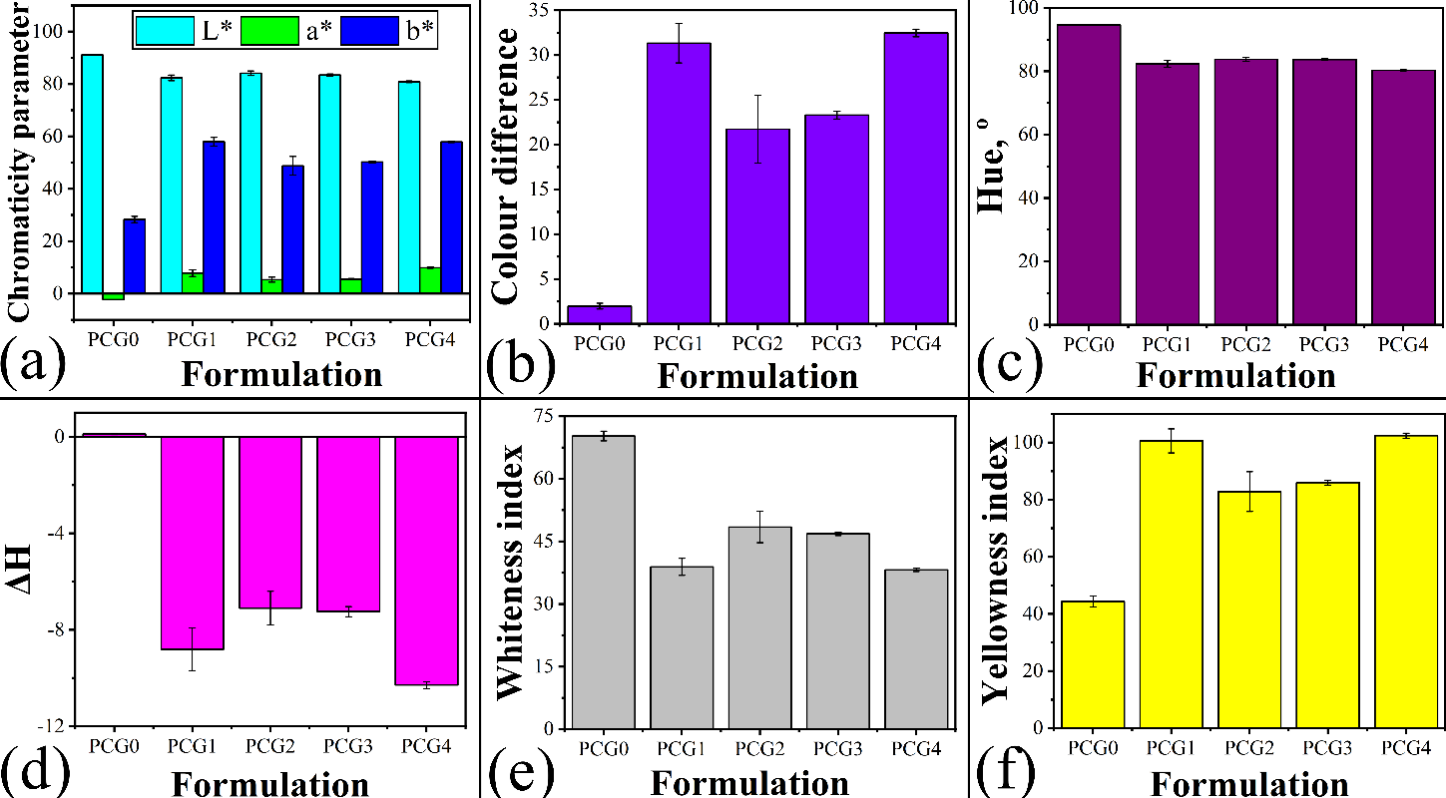
Colour parameter of the fabricated PVA/CS/GG composite film formulations: (a) chromaticity parameters, (b) colour difference, (c) hue angle, (d) hue difference, (e) whiteness index and (f) yellowness index

To improve the interpretation of visual colour inspection, colour parameters L*, a* and b* were measured using a colourimeter. Colour difference was calculated using Equation (3). The lightness index (L*) decreased upon the addition of gum ghatti to the polymer films. The L* parameter ranged from 0 (black) to 100 (white), while a* and b* varied from +120 to -120 [32]. In the control film, chromaticity parameters (a* and b*) showed green and yellow hues within the formulations. The addition of gum ghatti introduced red and yellow hues into the system (Figure 5a). This observation was confirmed by visual inspection. Colour differences greater than three are noticeable to the naked eye. Except for the control film, the colour difference was higher in GG films. The differences were visible to the naked eye, and the colourimetry results support this. The maximum colour difference was observed in PCG4. The whiteness index was highest in PCG0 and lowest in PCG4. The order of WI was PCG0 > PCG2 > PCG3 > PCG1 > PCG4. The highest yellowness index was observed in the PCG4 formulation. The sequence of YI was PCG4 > PCG1 > PCG3 > PCG2 > PCG0. The analysis showed that as YI increased, WI decreased. The hue angle and hue difference (ΔH) are shown in Figure 5c and 5d, respectively. The hue angle exceeded 90° in the chitosan-containing film. The hue angle decreased with the addition of gum ghatti, possibly due to the red-yellow hues within the system. This increased the yellowness index, while the decrease in hue angle may be linked to the red hues of gum ghatti.

### Swelling profile

**Figure 6.**
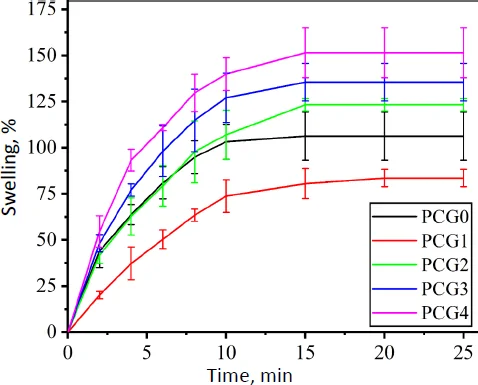
Swelling profile of the fabricated PVA/CS/GG ocular composite

Swelling plays a vital role in releasing the drug from the formulation to the active sites. The bioadhesive strength of the polymer was assessed by its swelling capacity. After reaching a critical point, polymer swelling also decreases its adhesive strength and reduces detachment at the polymer/tissue interface [33]. The presence of gum ghatti enhances the swelling properties of the fabricated ocular film. Mittal et al. prepared a gum ghatti-based hydrogel and reported that the porous structure of gum ghatti retains a large amount of water, thereby increasing swelling [34]. The swelling ability of the ocular composite film was found to follow the order of PCG4 > PCG3 > PCG2 > PCG0 > PCG1 (Figure 6). The decrease in swelling may be due to the strong interactions and bond formation among chitosan, PVA, and gum ghatti, which stabilize the structure and reduce the film's capacity to swell upon contact with water [24,35].

### X-ray diffraction analysis

**Figure 7.**
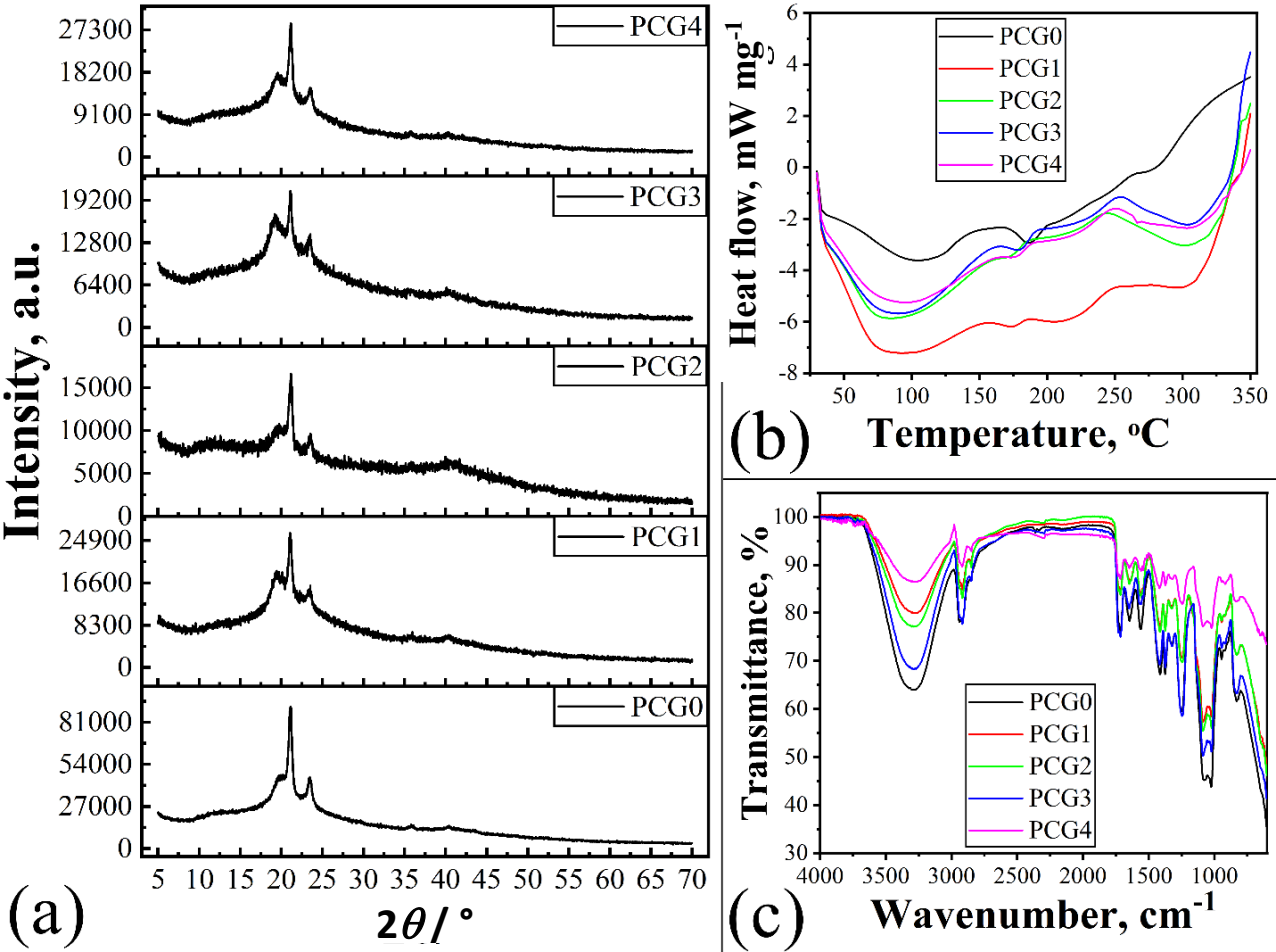
Molecular analysis of the composite films: (a) XRD analysis, (b) DSC analysis and (c) FTIR interaction

XRD analysis of the fabricated composite is shown in Figure 7a.

The pure drug moxifloxacin exhibits characteristic peaks at 2*θ* = 8.5, 10.05, 14.4, 16.95 and 17.35° [36]. The intense peak present in the spectra suggested the crystalline nature of the drug molecule. In all formulations, the absence of such peaks indicates that the drug molecules are amorphous within the fabricated composite films. Increasing the concentration of gum ghatti decreased the peak intensity, indicating lower crystallinity than in the control (PCG0). An intense peak is observed at 2*θ* = 22.7°, indicating the partially crystalline nature of PVA, consistent with the findings of Wen *et al.* [35]. An amorphous peak at 2*θ* = 23.2° indicates the amorphous nature of chitosan, as reported by Waly *et al.* [37]. To better understand the results, the amorphous and partially crystalline peaks were deconvoluted using Origin 2021 and are shown in Table 3. The average crystallite size ranked as PCG0 > PCG4 > PCG3 > PCG1 > PCG2 > PCG2.

**Table 3.** Parameters obtained from the deconvolution of XRD

Formulation/peak		2*θ* / °	FWHM (2*θ* / °)	d spacing, nm	Crystallite size, nm	Lattice strain	Dislocation density, m^-2^)
PCG0	Peak 1	19.12	15.57	0.464	0.54	0.40	3.43
	Peak 2	20.03	2.06	0.443	4.09	0.05	0.06
	Peak 3	21.10	0.45	0.421	18.58	0.01	0.00
	Peak 4	21.52	5.22	0.413	1.62	0.12	0.38
	Peak 5	23.47	0.63	0.379	13.35	0.01	0.01
	Average		4.79	0.424	7.64	0.12	0.78
PCG1	Peak 1	17.99	6.00	0.493	1.40	0.17	0.51
	Peak 2	20.65	3.42	0.430	2.47	0.08	0.16
	Peak 3	23.44	1.01	0.379	8.37	0.02	0.01
	Peak 4	25.11	3.40	0.354	2.50	0.07	0.16
	Average		3.46	0.414	3.69	0.08	0.21
PCG2	Peak 1	16.99	15.09	0.521	0.56	0.44	3.19
	Peak 2	20.18	2.67	0.440	3.16	0.07	0.10
	Peak 3	21.51	1.66	0.378	5.10	0.04	0.04
	Average		6.47	0.446	2.94	0.18	1.11
PCG3	Peak 1	15.44	2.31	0.574	3.63	0.07	0.08
	Peak 2	20.06	4.72	0.442	1.79	0.12	0.31
	Peak 3	23.47	0.75	0.379	11.23	0.02	0.01
	Peak 4	25.14	4.16	0.354	2.04	0.08	0.24
		Average	2.99	0.437	4.67	0.07	0.16
PCG4	Peak 1	19.94	6.36	0.445	1.32	0.16	0.57
	Peak 2	23.53	0.63	0.378	13.44	0.01	0.01
	Peak 3	25.46	2.74	0.350	3.11	0.05	0.10
		Average	3.24	0.391	5.96	0.07	0.23

### DSC thermogram

In the literature, moxifloxacin has been reported to exhibit a sharp endothermic peak at 239 °C [26,36,38]. The DSC thermogram of the ocular-fabricated film is shown in Figure 7b. A broad peak was observed in the temperature range of 50 to 120 °C, suggesting the presence of moisture in the composite film and the evaporation of water molecules. A small hump was observed near the temperature range of 180 to 190 °C, indicating the melting point of poly (vinyl) alcohol. A broad peak appeared above 290 °C, indicating the presence of GG, which stabilises the drug and polymer present in the formulation. The same type of observation was reported by Cheng *et al.* [39]. In all formulations, no intense endothermic peaks were observed around 239 °C, suggesting that the drug molecule is amorphous. Furthermore, the XRD analysis corroborated these data.

### FTIR analysis

The composition and interaction in the PVA/CS/GG ocular films were analysed using FTIR spectroscopy and are represented in Figure 7c. A broad peak was observed in the wavenumber range of 3650 to 3000 cm^-1^, indicating -OH stretching in the polymers. PVA, CS, and GG contain several functional groups, primarily -OH, -NH_2_, and carbohydrates, that participate in hydrogen bonding. A prominent peak was observed in the spectra of all films at 2912 cm^-1^, corresponding to the asymmetric C-H stretching vibration of the -CH_2_ groups. The peak appears across all formulations. Additionally, the peaks at 1643, 1419 and 1089 cm^-1^ correspond to C=O stretching, C-C bending, and C-O-C stretching vibrations, respectively [40]. The peak at 1717 cm^-1^ signifies the C=O acetate group of PVA, recognized as the characteristic peak of the PVA molecule. Peaks at 1643 and 1573 cm^-1^ represent the C=O bond of the amide I group of CONHR and N-N bending in the primary amine group of the amide II band [41]. A peak near 1259 cm^-1^ may be due to amide III vibrations of PVA or to interactions between the polymers. A shoulder peak around 1020 cm^-1^ indicated the C-O stretching vibration of PVA, CS and GG mixture. The final peak at approximately 813 cm^-1^ corresponds to C-C stretching in the CS molecule and its intensity diminishes as the GG concentration increases.

### In vitro mucoadhesion and puncture strength

Adhesiveness is a crucial parameter, especially for ocular formulations, and is particularly important when the formulation is applied to the anterior part of the eye. The release and permeation of the drug through the ocular barrier becomes possible when the contact time of the formulation increases. Blinking is the most challenging aspect of ocular drug delivery because it requires a force of 0.2 N. A polymeric film with a low peak adhesive force (PAF) of 0.2N can be easily repelled by tear fluid and blinking action [32]. Table 4 shows the mucoadhesion and puncture strength of the fabricated formulations. The highest PAF was found in formulation PCG3 (4.96 N). Increasing GG concentration generally increased the PAF, but at a 10 % GG concentration, the adhesion decreased. Several reports indicate that GG can inhibit adhesion at a concentration of 10 % [42]. Nagamadhu *et al.* [43] also reported that GG loses its original properties after a 10-fold increase in concentration. In the 10 % (PCG4) formulation containing GG, the lowest PAF and total work of adhesion (TWA) were observed. Similar trends were observed in puncture strength: initially, the breaking force increased, whereas in PCG4 it decreased. This suggests that the threshold for GG is around 10 %.

**Table 4.** *In vitro* mucoadhesion and puncture strength

Formulation	Parameter			
	PAF, N	TWA, N s^-1^	Cohesiveness, mm	Puncture strength, g m
PCG0	4.13	9.52	1.31	4121.00
PCG1	4.04	7.02	1.90	4781.00
PCG2	4.23	12.37	1.13	5289.00
PCG3	4.96	9.25	1.774	5631.00
PCG4	3.20	2.27	1.66	5170.00

### In vitro drug diffusion study

**Figure 8.**
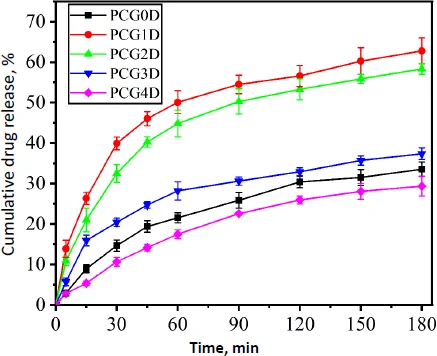
*In vitro* drug release from the composite

Release of moxifloxacin from the composite was evaluated using a Franz diffusion cell. The drug diffusion study was conducted for 180 min, and the release pattern of the composite is shown in Figure 8. After 180 minutes, the release order was PCG1D > PCG2D > PCG3D > PCG0D > PCG4D. It is observed that as gum ghatti concentration in the composite increases, drug release decreases. This release pattern correlates with the swelling profile. The greater the swelling, the lower the drug release from the formulations [25].

The release parameter was also fitted to various mathematical models and is tabulated in Table 5. The correlation coefficient (*R*^2^) ranged from 0.61 to 0.92 for 1^st^ order release kinetics. PCG3D was not a good fit for the 1st-order model, whereas PCG1D was the best fit. Within the first 15 minutes, the maximum release was observed in the PCG1D formulations, whereas the lowest release was observed in the PCG4D formulations. The same trend was observed using the Higuchi fractal dimension, with PCG1D > PCG2D > PCG3D > PCG0D > PCG4D.

**Table 5.** Model parameter of drug diffusion study

Model	Release parameters	PCG0D	PCG1D	PCG2D	PCG3D	PCG4D
1^st^ order	*K*_1_	0.003 ± 0.000	0.0009 ± 0.001	0.007 ± 0.001	0.004 ± 0.000	0.002 ± 0.000
	*R* ^2^	0.829 ± 0.017	0.929 ± 0.011	0.745 ± 0.038	0.611 ± 0.035	0.899 ± 0.042
Higuchi	*K*_H_	2.636 ± 0.161	5.418 ± 0.255	4.933 ± 0.208	3.107 ± 0.123	2.232 ± 0.112
	*R* ^2^	0.982 ± 0.004	0.887 ± 0.019	0.936 ± 0.019	0.938 ± 0.011	0.974 ± 0.006
Korse Meyer-Peppas	*K*_P_	2.665 ± 0.364	12.093 ± 1.017	8.805 ± 1.311	5.586 ± 0.606	1.531 ± 0.307
	*n*	0.499 ± 0.017	0.327 ± 0.012	0.376 ± 0.025	0.374 ± 0.016	0.584 ± 0.049
	*R* ^2^	0.983 ± 0.004	0.985 ± 0.004	0.974 ± 0.003	0.976 ± 0.001	0.984 ± 0.007
Peppas-Shalin	*K*_d_	1.292 ± 0.223	7.035 ± 0.754	4.588 ± 0.526	3.401 ± 0.524	-1.971 ± 4.501
	*K*_r_	-0.013 ± 0.004	0.203 ± 0.034	-0.093 ± 0.019	-0.079 ± 0.020	1.994 ± 3.460
	*m*	0.747 ± 0.027	0.556 ± 0.018	0.625 ± 0.009	0.579 ± 0.030	0.650 ± 0.381
	*R* ^2^	0.996 ± 0.001	0.993 ± 0.002	0.996 ± 0.002	0.991 ± 0.002	0.997 ± 0.002

*K_1_* represents the first-order release rate constant, *K*_H_ represents the Higuchi rate constant, *K*_p_ signifies the release rate constant, *n* describes the diffusion exponent, *K*_d_ is defined as the Fickian diffusion constant, *K*_r_ is defined as the case-II relaxation constant, and m is defined as the exponent of diffusion.

All formulations exhibited correlation coefficients greater than 0.88. PCG1D, PCG2D, and PCG3D exhibited Fickian drug release, whereas PCG0D and PCG4D showed non-Fickian or anomalous drug diffusion [44]. In both the KP and PS models, an excellent correlation was observed, with values exceeding 0.98.

This suggested that there was no significant difference between the observed and predicted data. Higher swelling takes a longer pathway for the movement of the drug, causing a delay in drug release. The crystal sizes were in the order PCG0 > PCG4 > PCG3 > PCG1 > PCG2. The release profile can also be related to the crystal size of the formulation molecule. The smaller the crystal size, the higher the drug release and vice versa. The largest crystal size was observed in the GG-incorporated film, PCG4 (5.96), which also showed the lowest drug release.

### In vitro antimicrobial efficacy

**Figure 9.**
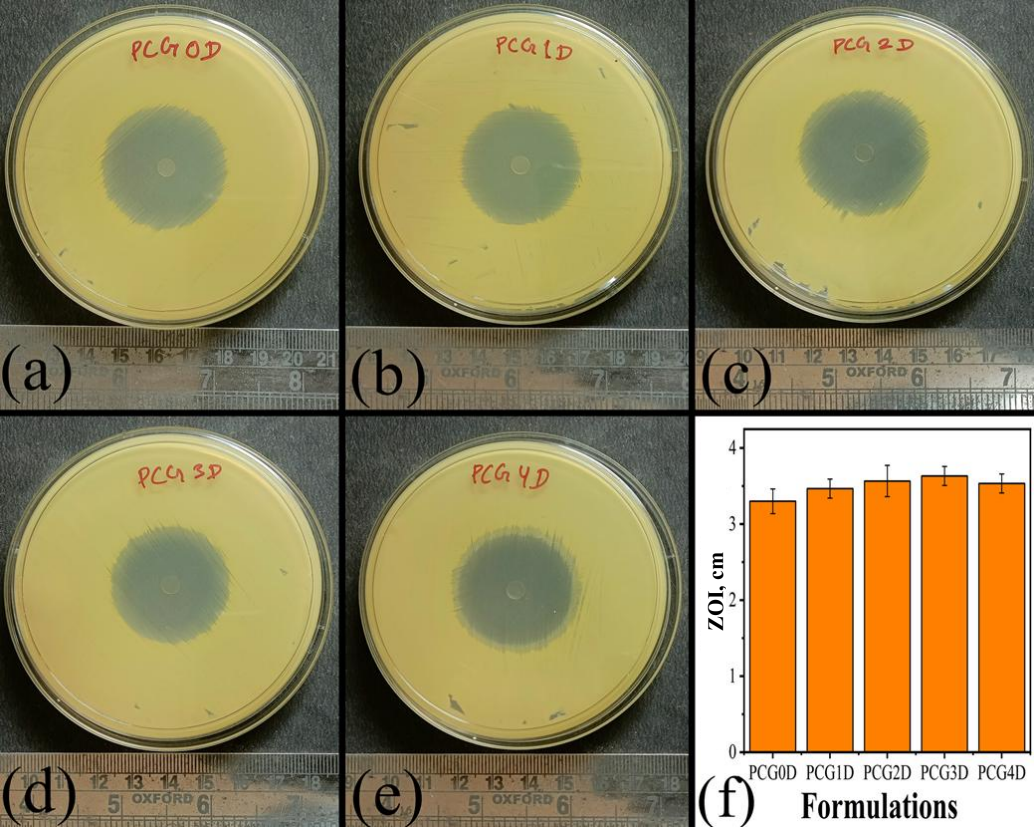
*In vitro* antibacterial activity against *Pseudomonas aeruginosa*. (a) PCG0D, (b) PCG1D, (c) PCG2D, (d) PCG3D, (e) PCG4D and (f) bar diagram representing ZOI against *P. aeruginosa*

**Figure 10.**
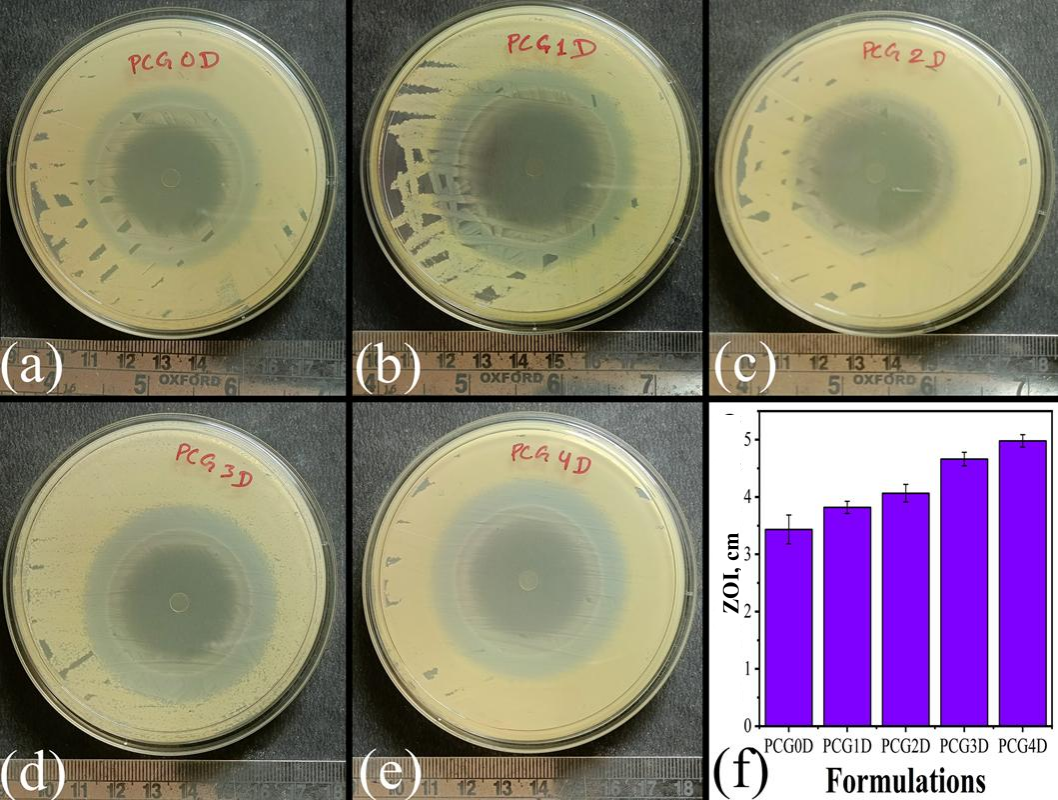
*In vitro* antibacterial activity against *Staphylococcus aureus*. (a) PCG0D, (b) PCG1D, (c) PCG2D, (d) PCG3D, (e) PCG4D and (f) bar diagram representing ZOI against *S. aureus*

The antimicrobial efficacy of the fabricated formulation was tested against both microorganisms. *Pseudomonas aeruginosa* and *Staphylococcus aureus* were used as Gram-negative (Figure 9) and Gram-positive (Figure 10) organisms, respectively, against the fabricated formulations. Currently, several studies indicate that *Pseudomonas aeruginosa* contributes to corneal ulcers, keratitis, and endophthalmitis [45].

Moxifloxacin is a fourth-generation antibacterial drug that inhibits the DNA gyrase of bacteria. Additionally, the drug has candidacidal properties, appears to inhibit DNA topoisomerase II in *C. albicans*, and interferes with the yeast-to-hyphal transition by affecting key signalling pathways. Moxifloxacin inhibits the cell cycle of *Candida albicans* during S phase, thereby demonstrating its antifungal activity [45,46].

The results showed that ZOI increased with increasing GG concentration. This may be due to the prolonged drug release, which inhibits bacterial growth for a longer period. The diffusion of the drug in the agar media kills the bacteria, resulting in a clear zone. In PCG4D, the ZOI decreased, but remained higher than in the control sample (PCG0D). This may be due to a defect in the crystal size. The ZOI was found in the order of PCG3D (3.63±0.12) > PCG2D (3.57±0.21) > PCG4D (3.57±0.21) > PCG1D (3.47±0.12) > PCG0D (3.30±0.16) (Figure 5). Moxifloxacin indicates that Gram-positive bacteria lack an outer membrane and have only a thick peptidoglycan layer. Thus, the drug's penetration was easier than that of Gram-negative bacteria (*P. aeruginosa*) [36]. Compared with *P. aeruginosa*, the drug showed a greater ZOI against *S. aureus*. The ZOI was found in the order of PCG4D > PCG3D > PCG2D > PCG1D > PCG0D. It can be concluded that a prepared ocular composite can inhibit both types of bacteria and control the different ocular diseases.

### In vitro cell biocompatibility assay

Before instilling the formulation into the cul-de-sac region of the eye, a cell compatibility study was conducted using ocular cells. The control formulation exhibited a cell viability of 87 %, whereas the formulation containing GG showed a viability greater than 89 %. To formulate the ocular composite, PVA, CS, and GG are used. All the polymers are widely used for ocular drug delivery, are FDA-approved, and are listed on the GRAS (Generally Recognized as Safe) list. According to ISO guidelines, cell viability greater than 70 % can be considered safe for topical use, particularly for corneal delivery. So, the prepared formulation can be applied to the corneal surface for 3 days without causing toxicity [32].

### In vivo ocular irritation test

**Figure 11.**
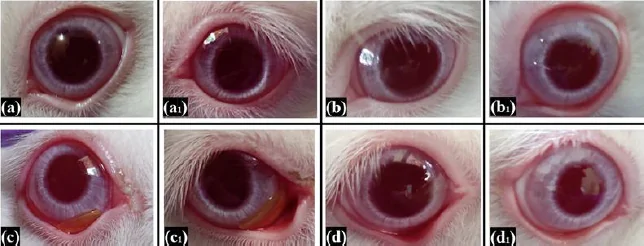
Ocular irritation study (a) left eye injected with ATF (a, b, c, d) and the right eye was instilled with ocular composite (a_1_, b_1_, c_1_, d_1_) at 0 hour (a, a_1_), 12 hours (b, b_1_), 24 hours (c, c_1_), and 48 hours (d, d_1_), respectively

An *in vivo* ocular irritation study was conducted to assess swelling, redness, and inflammation following instillation of the composite film formulations. The experiment was carried out for 48 h. At different time points, pictographs were obtained and evaluated for corneal swelling, inflammation, redness, and ocular chemosis (Figure 11). In the ocular irritation test, no ocular chemosis was observed after 48 hours, suggesting that the prepared formulations are safe for corneal delivery.

### In vivo anti-inflammatory activity and corneal integrity analysis

**Figure 12.**
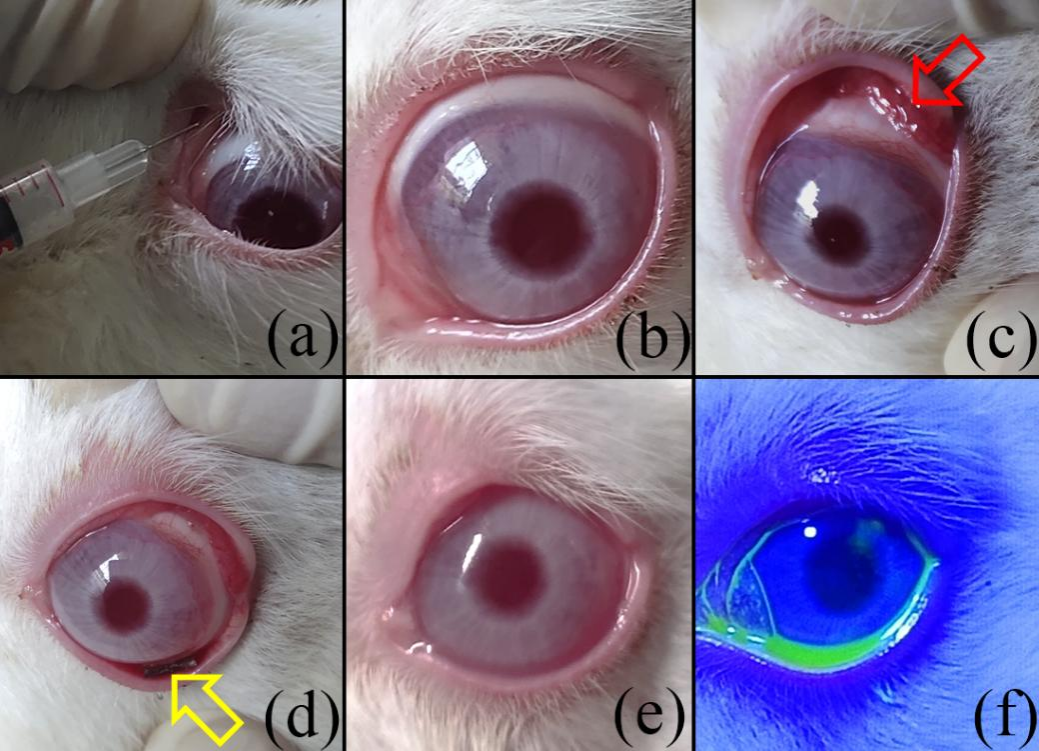
Anti-inflammatory and corneal integrity study of the developed ocular film formulations. (a) injecting carrageenan to develop corneal inflammation, (b) normal eye of the albino rabbit, (c) inflammation developed after inducing carrageenan, (d) diminishing of corneal inflammation with 1.5 h after inserting the prepared composite film, (e) recovery eye after inflammation and (f) corneal integrity of the Rabbit eye with sodium fluorescein dye

In vivo anti-inflammatory effects on the corneal surface were induced by carrageenan injection. Carrageenan is an endotoxin, and the induction of carrageenan dilated the conjunctival blood vessels present in the cornea. It used to produce local inflammation. Moxifloxacin is a USFDA-approved medication used to treat pink eye or inflammation triggered by allergens [47]. Figure 12a represents the insertion of the carrageenan solution into the subconjunctival region of the rabbit eye. Inflammation is observed 30 minutes after carrageenan injection (Figure 12c).

After inducing inflammation, a prepared, sterilized ocular film was inserted into the cul-de-sac of the eye (Figure 12d). Within 1 h, inflammation had vanished (Figure 12e). After the inflammation resolved, 0.5% moxifloxacin eye drops (MOXITOR) were used to prevent contamination. Redness and inflammation persist for 2 hours in the no-treatment group. After six hours of inflammation, sodium fluorescein dye was used to evaluate corneal integrity in the rabbit eye. The dye was inserted in the cul-de-sac region (Figure 12f). No dark spot was found in the corneal region, confirming there was no coagulant or thrombosis of the blood occurring in the eye. Thus, it can be assumed that the prepared formulation has potential for the treatment of ocular conjunctivitis and can be safely used for pink eye, bacterial conjunctivitis, or allergic conjunctivitis.

## Conclusions

The solvent evaporation method is a traditional approach for preparing polymeric films. A poly (vinyl alcohol)/chitosan/gum ghatti-based polymeric ocular composite was fabricated using this method to prevent ocular conjunctivitis, pink eye, bacterial conjunctivitis, and allergic inflammation. GTA was used as a crosslinker at a very low concentration, and free GTA was neutralized by soaking in a 0.1 M glycine solution for 4 hours to prevent ocular chemosis or irritation. Thickness increased with higher GG concentration. A pH range of 5.5 to 7.5 was found to be safe for ocular delivery. The film's opacity increased, indicating reduced transparency. All formulations exhibited haemolysis rates below 5 %, indicating safety for corneal delivery. Microscopic analysis revealed that the incorporation of GG and the chitosan molecular flexibility would be diminished within the polymer. The addition of gum ghatti imparts a red and yellow hue to the system. The presence of gum ghatti enhances the swelling properties of the fabricated ocular film. XRD and DSC analyses revealed that the drug in the formulation is amorphous. The presence of GG stabilized both the drug and the polymer in the formulation. There is no molecular interaction between the drug and polymer, indicating compatibility within the formulations. Increasing GG concentration elevated PAF; however, at 10 % GG, adhesion was reduced, indicating a critical threshold. The incorporation of GG reduced drug release by inhibiting crystalline defects and thereby supporting the swelling profile. Drug release followed both Fickian and non- Fickian pathways. The drug-release model showed a strong correlation between observed and predicted data. A clear zone of inhibition was observed against both Gram-negative (*Pseudomonas aeruginosa*) and Gram-positive (*Staphylococcus aureus*) bacteria. Cell viability exceeded 87 %, indicating that the formulation can be used for topical application for more than 72 hours. An in vivo ocular irritation study indicated the formulations are safe for corneal delivery, with no signs of swelling, inflammation, redness, or chemosis. Carrageenan-induced in vivo anti-inflammatory tests showed reductions in redness and inflammation within 1.5 hours. Assessment of corneal integrity revealed no dark spots, confirming that no coagulant or thrombosis occurs in the eye. Thus, the formulation shows potential for the treatment of ocular conjunctivitis and can be safely used for pink eye, bacterial conjunctivitis, or allergic conjunctivitis.

## Supplementary material

Additional data are available at https://pub.iapchem.org/ojs/index.php/admet/article/view/3200, or from the corresponding author on request. The supplementary materials contain a detailed description of the physicochemical properties of the material and the methods section.


