# Supplementary material for: Enhanced anti-inflammatory and antibacterial efficacy of a novel gum ghatti-infused polymeric film for ocular drug delivery
Source: ADMET DMPK. 2026 May 28;14:3200. doi: 10.5599/admet.3200 (PMC13499658; doi:10.5599/admet.3200)
Supplement: Supplementary file 1 [file ADMET-14-3200-S1.pdf]

ADMET & DMPK **14** (2026) S3200

Open Access : ISSN : 1848-7718
http://www.pub.iapchem.org/ojs/index.php/admet

Supplementary material to

Enhanced anti-inflammatory and antibacterial efficacy of a novel gum ghatti-infused polymeric film for ocular drug delivery

Swagatika Das[1], Sk Habibullah[2], Yashwant Giri[1], Amulyaratna Behera[3], Gurudutta Pattnaik[1] and Biswaranjan Mohanty[3]

[1]*Department of Pharmaceutics, School of Pharmacy and Life Sciences, Centurion University of Technology and Management, Odisha 752050, India*
[2]*Department of Pharmaceutics, University Department of Pharmaceutical Sciences, Utkal University, Vani Vihar, Bhubaneswar, Odisha 751004, India*
[3]*Department of Pharmaceutics, School of Pharmacy, DRIEMS University, Tangi, Cuttack, Odisha 754022, India*

ADMET & DMPK **14** (2026) 3200; https://doi.org/10.5599/admet.3200

Materials and methods

Physicochemical properties

Physicochemical properties such as weight uniformity, ocular composite thickness, surface pH, opacity, folding endurance, and blood biocompatibility were measured. The fabricated ocular composite was cut into a 1×1 cm^2 piece, weighed with a digital balance (Wensar, LPB21), and the weight was recorded. Five or six random portions of the film were selected, and the thickness was measured using a digital micrometer (Mitutoyo, Japan). The 1×1 cm^2 films were immersed in a petri dish containing 5 ml of double-distilled water. After 10 minutes, the water's pH was measured with a pH meter (L1617, Elico, Mumbai, India). To assess opacity, the film was cut into 40×50 mm pieces, and absorbance was recorded at 600 nm using a UV-Visible spectrophotometer (Shimadzu, Japan). Opacity was calculated using article Equation (1). Folding endurance was tested until a 2×2 cm^2 film was broken. Blood containing sodium citrate was used to evaluate hemolysis. In brief, 4 mL of sodium citrate blood was mixed with 5 mL of normal saline. Negative control consisted of 0.5 mL citrated blood with 9.5 mL normal saline; positive control contained 0.5 mL 0.1 M HCl, 0.5 mL blood, and 9 mL normal saline; and the test samples were prepared from previously collected lechate (incubated for 1 hour at 37 °C; 9 mL normal saline with 0.5 mL citrated blood and 0.5 mL lechate). All samples were incubated again for 1 hour at 37 °C, then centrifuged at 300 rpm for 10 minutes. The supernatant's absorbance was measured spectrophotometrically at 545 nm, and coagulation was calculated using article Equation (2)[1,2]. All physicochemical properties were expressed as mean ± SD (n = 6).

Drug release profile

Diffusion of the drug was assessed through a Franz diffusion cell. 12 ml of artificial tear fluid (ATF, composition: NaCl 6.8 g, $NaHCO_3$ 0.22 g, $CaCl_2 \cdot 2H_2O$ 0.08 g, KCl 1.4 g added to deionised water and volume made up to 1000 mL) [3]. The donor and receiver compartment of the Franz cell was separated by a semipermeable dialysis membrane (MW: 60 kDa). A film (10 mm in diameter) was placed over the pre-hydrated dialysis membrane. Receptor compartment fluid temperature was maintained at 37 °C throughout the experiment. Stirring speed was maintained at 100 rpm with the help of a magnetic stirrer. The area of diffusion was 0.64 cm^2. At a predetermined interval, 1 mL of the sample was removed and replaced with fresh ATF. Presence of the drug in the sample was measured through a UV-vis spectrophotometer (UV-1900i,

Shimadzu Corporation, Japan) at 289 nm. Different mathematical models like 1st order, Equation (S1); Higuchi, Equation (S2); Korsmeyer-Peppas, Equation (S3) and Peppas-Shalin, Equation (S4) were fitted to the observed data.

$$\frac{M_\infty}{M_\mathrm{i}} = m = 100\left(1\text{-}\mathrm{e}^{-K_1 t}\right) \tag{S1}$$

where M_∞ is amount of drug at the equilibrium state, M_i is amount of drug released over time *t*, *m* is fraction of the solute released, K_1 is release rate constant and t = time.

$$\frac{M_\infty}{M_\mathrm{i}} = m = k t^{0.5} \tag{S2}$$

where *k* is release rate constant.

$$\frac{M_\infty}{M_\mathrm{i}} = m = k t^{n} \tag{S3}$$

where *n* is the diffusion exponent,

$$\frac{M_t}{M_\infty} = k_1 t^m + k_2 t^{2m} \tag{S4}$$

where k_1, k_2 and *m* are constants, $k_1 t^m$ is Fickian diffusional contribution and $k_2 t^{2m}$ is case-II relaxational contribution

References

[1] S. Habibullah, J.R. Meher, M. Das, T. Das, R. Swain, B. Mohanty, S. Mallick. Moxifloxacin in HPMC-nanocellulose composite film for the management of ocular inflammation: Effect of carboxymethylated gum on permeation and antimicrobial activity. *International Journal of Biological Macromolecules* **310** (2025) 143302. https://doi.org/10.1016/j.ijbiomac.2025.143302

[2] S. Habibullah, M. Das, S. Ghosh, T. Das, R. Swain, B. Mohanty, S. Mallick. Effect of beeswax-cottonseed oil based oleogel contribution in nanocellulosic bigel for ophthalmic moxifloxacin delivery. *Journal of Drug Delivery Science and Technology* **112** (2025) 107287. https://doi.org/10.1016/j.jddst.2025.107287

[3] Y. Liu, J. Liu, X. Zhang, R. Zhang, Y. Huang, C. Wu. *In situ* gelling gelrite/alginate formulations as vehicles for ophthalmic drug delivery. *AAPS PharmSciTech* **11(2)** (2010) 610-620. https://doi.org/10.1208/s12249-010-9413-0

CC BY
